# Association Between Weight-Adjusted Waist Index (WWI) and Bone Mineral Density in Postmenopausal Women: A Cross-Sectional Analysis of NHANES Data

**DOI:** 10.1155/ije/6618917

**Published:** 2025-09-27

**Authors:** Qifeng Jing, Yan Tan

**Affiliations:** ^1^Department of Radiology, The First People's Hospital of Xiaoshan District, Xiaoshan Affiliated Hospital of Wenzhou Medical University, Hangzhou, Zhejiang, China; ^2^Department of Electrocardiogram, The First People's Hospital of Xiaoshan District, Xiaoshan Affiliated Hospital of Wenzhou Medical University, Hangzhou, Zhejiang, China

**Keywords:** bone mineral density, NHANES, osteoporosis, postmenopausal women, weight-adjusted waist index

## Abstract

**Background:** Osteoporosis represents a critical public health challenge, particularly among postmenopausal women, characterized by reduced bone mineral density (BMD) and increased fracture risk. Traditional obesity metrics such as body mass index (BMI) and waist circumference (WC) have limitations in assessing bone health due to their inability to differentiate lean and fat mass. The weight-adjusted waist index (WWI), a novel anthropometric indicator, offers a more nuanced approach to evaluating body composition and metabolic risks.

**Objective:** The WWI is a novel obesity metric that demonstrates superior accuracy in evaluating both muscle mass and adiposity compared to conventional anthropometric measurements. This cross-sectional study examined the relationship between WWI and BMD at the femoral neck in a nationally representative sample of American postmenopausal women.

**Methods:** A cross-sectional analysis was conducted using data from the National Health and Nutrition Examination Survey (NHANES), including 3198 postmenopausal women aged 40 and older. WWI was calculated by dividing WC by the square root of body weight. BMD was assessed using dual-energy X-ray absorptiometry (DXA). Multivariate regression analyses were performed, adjusting for potential confounders including age, race, height, BMI, and metabolic markers.

**Results:** Multiple regression analyses revealed a significant negative correlation between WWI and femoral neck BMD. A critical threshold of 10.32 was identified, beyond which the relationship with BMD shifted. Below this threshold, higher WWI levels showed a protective effect on BMD (*β* = 0.03, *p*=0.0265), while above the threshold, WWI exhibited a significant negative influence (*β* = −0.01, *p* < 0.0001). Stratified analyses demonstrated variations in the WWI-BMD relationship across different subgroups, including age, race, and menopause status.

**Conclusion:** This cross-sectional analysis revealed a significant negative correlation between the WWI and femoral neck BMD in a nationally representative cohort of American postmenopausal women. Notably, the association demonstrated a nonlinear inverted U-shaped pattern with an identifiable threshold effect.

## 1. Background

Osteoporosis, characterized by reduced bone mineral density (BMD) and increased fracture risk, represents a critical public health burden, particularly among postmenopausal women, with estrogen deficiency significantly accelerating bone loss [1, 2]. Traditional obesity metrics such as body mass index (BMI) and waist circumference (WC) have been extensively studied for their associations with BMD; however, these measures have limitations due to their failure to differentiate between lean mass and fat mass, which raises concerns about their reliability in assessing bone health [3, 4]. The weight-adjusted waist index (WWI), a novel anthropometric indicator that integrates weight and height, aims to better reflect central obesity and associated metabolic risks compared to conventional indices [5, 6]. Preliminary findings show that WWI has demonstrated superior performance in predicting cardiovascular and metabolic disorders, suggesting its potential utility in interdisciplinary health assessments [7, 8].

Emerging evidence suggests that fat distribution and metabolic dysfunction, rather than total adiposity alone, may exert differential effects on bone remodeling. Recent studies emphasize that localized fat distribution, especially visceral fat, negatively impacts bone health, independent of overall body weight. For instance, investigations highlight how metabolic dysfunction can lead to a state where increased fat mass does not equate to improved BMD, particularly in populations such as postmenopausal women who experience hormonal changes affecting bone density [9, 10]. Despite the significant implications of these findings, studies evaluating the WWI, which emphasizes the waist-to-weight ratio, in the context of osteoporosis are scarce. This oversight in research prompts a critical examination of WWI's prognostic value for skeletal health, particularly in light of contemporary understandings of how body composition affects osteoporosis risk [11].

The National Health and Nutrition Examination Survey (NHANES) provides a unique opportunity to address this gap in research literature. NHANES offers nationally representative data alongside standardized dual-energy X-ray absorptiometry (DXA) measurements for assessing BMD, as well as detailed anthropometric records that could facilitate robust analysis of WWI's role in bone health [12, 13].

This study aims to investigate the association between WWI and BMD in postmenopausal women using NHANES data, hypothesizing that higher WWI values correlate with lower BMD, independent of traditional obesity indices. By elucidating WWI's utility as a predictor of skeletal deterioration, this research seeks to advance understanding of body composition metrics in osteoporosis risk stratification, offering insights for targeted screening and preventive strategies in a high-risk population.

## 2. Methods

### 2.1. Survey Description and Study Participants

**Figure 1:**
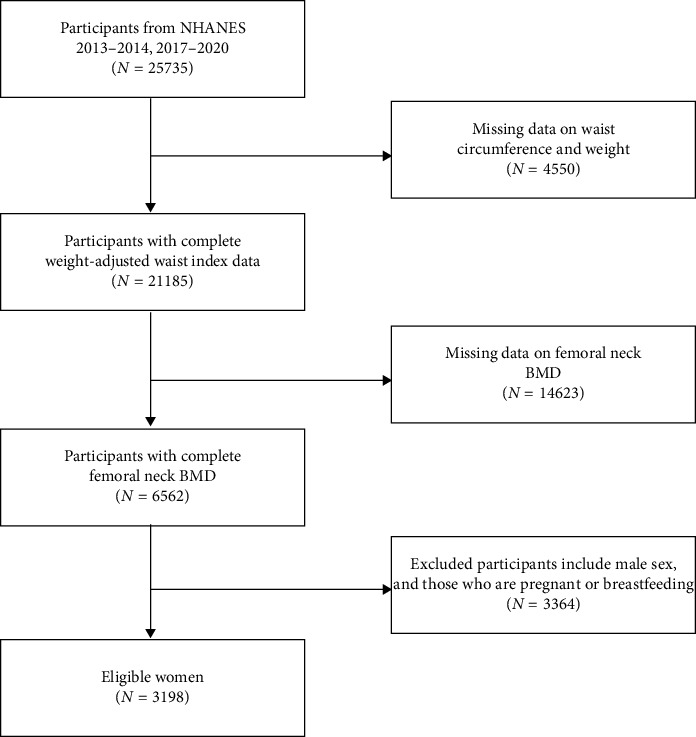
Flowchart of the sample selection.

The National Center for Health Statistics (NCHS) is conducting a nationally representative cross-sectional study, the NHANES [14]. This survey employs a stratified multistage probability sampling method to collect comprehensive data and is updated every 2 years. NHANES provides valuable secondary analysis data on health and nutrition in the United States. NHANES data can be accessed on the NHANES website (https://www.cdc.gov/nchs/nhanes.htm; accessed March 2, 2025). The NCHS Research Ethics Review Board has approved the NHANES research protocol, and written informed consent was obtained from participants before their involvement in the study [15]. The inclusion criteria for the NHANES 2013–2014 and 2017–2020 cohorts required participants to be postmenopausal women aged 40 and older, with complete data on WWI and femoral neck BMD. Exclusion criteria included pregnant and breastfeeding women. After applying the inclusion and exclusion criteria, the final number of participants in the study was 3198 (Figure 1).

### 2.2. Study Variables

The WWI is calculated by dividing the waist measurement in centimeters by the square root of body weight in kilograms, serving as a method to evaluate body fat and muscle mass. Participants' weights and waist measurements were recorded by certified health professionals at the mobile examination unit. By removing their shoes and heavy clothing, the participants' weights were assessed. To estimate the WC, a horizontal line was drawn above the highest lateral edge of the right iliac bone, and a tape measure was placed where the lines intersected [16]. Femoral neck BMD was assessed using a DXA scan. Covariates included age, income-to-poverty ratio (PIR), standing height, BMI, total femur BMD, femoral neck BMD, alkaline phosphatase (ALP), blood urea nitrogen, globulin, glucose, triglycerides, uric acid, race, education level, and menopause status. The menopausal status is confirmed through self-reported reproductive health questionnaires in the NHANES survey. Women are classified as postmenopausal if they answer “no” to the question, “Have you had a menstrual period in the past 12 months? (do not include bleeding due to medical conditions, hormone therapy, or surgery)” and select “hysterectomy” or “menopause/life changes” as the reason for not having menstrual periods. Detailed information about the questionnaire can be found in the NHANES database and is accessible through the official website.

### 2.3. Statistical Analysis

The statistical analyses followed NCHS guidelines, using sample weights and considering the intricate multistage cluster survey design. Participant demographics by WWI tertile were assessed using the weighted chi-square and *t*-tests. The analysis of collinearity was conducted to assess and remove variables with collinearity, where a variance inflation factor (VIF) greater than 10 was deemed collinear. In addition, the relationship between WWI and BMD was resolved in this study by employing smooth and generalized additive models. When nonlinearity is identified, the research will utilize a recursive algorithm to find the inflection point. Next, a two-stage piecewise linear regression model was utilized to study the connection between WWI and BMD on both sides of the inflection point. Subgroup analysis was carried out to investigate the relationship between WWI and BMD in various subpopulations based on factors such as age, race, standing height, BMI, and menopause status. Continuous variables with missing values were appropriately categorized into tertiles or quartiles based on their distributions, with missing observations assigned to a dedicated “Missing” category. Categorical variables with missing data were handled as a separate category to avoid bias from arbitrary classification. Sensitivity analyses were conducted to evaluate the potential impact of missing data on the results. The analyses were performed using R (https://www.Rproject.org) and EmpowerStats (https://www.empowerstats.com), with a *p* value of less than 0.05 deemed statistically significant.

## 3. Results

### 3.1. Baseline Characteristics

This study enrolled 3198 participants, divided into three tertiles (low, middle, and high), with 1066 individuals in each tertile.  Table 1 presents the baseline characteristics of the participants. Significant differences were observed across tertiles in terms of demographic characteristics, physical traits, and metabolic indicators. The high tertile had the oldest average age (65.33 ± 10.31 years), while the low tertile had the youngest (57.17 ± 10.53 years). Standing height decreased with tertile, with the high tertile being the shortest (156.63 ± 6.82 cm) compared to the low tertile (162.14 ± 6.47 cm). Conversely, BMI increased across tertiles, reaching 31.85 ± 6.66 kg/m^2^ in the high tertile, significantly higher than the low tertile's 26.41 ± 5.85 kg/m^2^. Femoral neck BMD was lowest in the high tertile (0.71 ± 0.14) compared to the low tertile (0.75 ± 0.14). Metabolic markers such as ALP, blood urea nitrogen, glucose, triglycerides, and uric acid were significantly higher in the high tertile. Race distribution and educational attainment also varied significantly across tertiles, with non-Hispanic Whites being the largest group in the high tertile (44.37%) and the highest proportion of participants with less than a high school education (28.08%). Menopause status differed notably, with the high tertile having the highest proportion of postmenopausal individuals (87.71%) compared to the low tertile (24.67%) (Table 1).

### 3.2. Association Between WWI and Femoral Neck BMD

**Figure 2:**
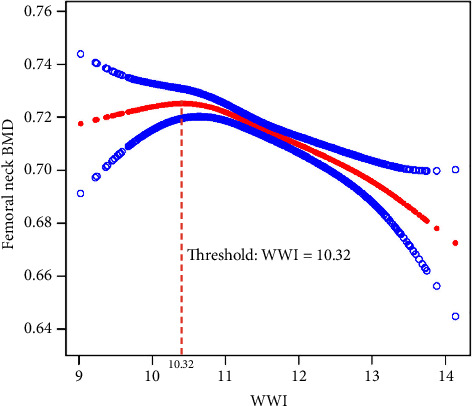
Association between weight-adjusted waist index (cm/√kg) and femoral neck BMD in postmenopausal women. Age, race, standing height, BMI, total femur BMD, ALP, BUN, globulin, serum glucose, triglycerides, and uric acid were adjusted (the solid red line indicates the smooth curve fit between variables, and the blue bands denote the 95% confidence interval from the fit).

Table 2 presents the connection between WWI and femoral neck BMD. The multiple regression analysis confirmed the significant impact of WWI on femoral neck BMD after adjusting for confounders. In the nonadjusted model, WWI showed a negative trend (*β* = −0.02, *p*=0.0003). After adjusting for age, race, standing height, and BMI (Adjust I), the negative association strengthened (*β* = −0.03, *p* < 0.0001). The Adjust II model, which included additional variables such as total femur BMD and metabolic markers, further confirmed the negative effect (*β* = −0.02, *p* < 0.0001) (Table 2 and Figure 2).

### 3.3. Threshold Effect Analysis

The threshold effect analysis (Table 3) identified a critical threshold of WWI at 10.32, beyond which the relationship with femoral neck BMD shifted. Below this threshold, higher WWI levels were associated with a protective effect on BMD (*β* = 0.03, *p*=0.0265). Above the threshold, WWI exhibited a significant negative influence (*β* = −0.01, *p* < 0.0001). The difference in effects across the threshold was statistically significant (effect difference = −0.04, *p*=0.0023), highlighting the nonlinear nature of WWI's impact on BMD.

### 3.4. Stratified Analysis

The stratified analysis revealed variations in the association between WWI and femoral neck BMD across subgroups. Age-stratified results showed a positive association in the 40–55 year-old group (*β* = 0.01, *p*=0.0773) and a negative association in the 66–80 year-old group (*β* = −0.01, *p*=0.0056). Racial differences were observed, with non-Hispanic Blacks having higher BMD compared to Mexican Americans (*β* = −0.00, *p*=0.8111), while other racial groups showed a mild negative association (*β* = −0.02, *p*=0.0055). Standing height and BMI categories indicated taller individuals, and those with higher BMI generally had better BMD, though BMI's positive effect diminished at higher levels. Menopause status also played a role, with premenopausal women showing higher BMD (*β* = 0.03, *p*=0.0016) and the “Other or refused” category presenting a notable negative effect (*β* = −0.06, *p* < 0.0001) (Table 4).

Supporting information containing additional threshold effect analyses (Supporting Table S1) and smoothed curve fitting results (Supporting Figure S1) are available online. These analyses further validate the robustness of the observed nonlinear relationship between WWI and femoral neck BMD.

## 4. Discussion

This study aimed to investigate the relationship between the WWI and BMD in postmenopausal women, utilizing data from the NHANES. A cross-sectional analysis was conducted, including 3198 eligible postmenopausal participants. We employed DXA to assess BMD and performed multiple regression analyses to control for confounding factors such as age, race, height, and BMI. Our findings revealed a significant negative correlation between WWI and femoral neck BMD, particularly pronounced when WWI exceeded a threshold of 10.32. This highlights WWI as a critical indicator for assessing skeletal health risks, suggesting that the complexities of body composition should be considered in screening and preventive strategies for high-risk populations.

The association between the WWI and BMD or osteoporosis has been explored in several studies, highlighting its significance across different age groups. One study demonstrated a negative correlation between WWI and BMD among adults, suggesting that higher WWI levels are associated with lower BMD, which could potentially increase the risk of osteoporosis [17]. This inverse relationship was also observed in adolescents, where a significant negative association between WWI and total BMD was identified, emphasizing the importance of monitoring WWI in younger populations to prevent future bone health issues [18]. In older adults, the relationship between WWI and BMD continues to be significant. A study involving older adults revealed that higher WWI scores were associated with increased odds of frailty, which is often linked to decreased bone density and higher osteoporosis risk [19]. This underscores the importance of considering WWI as part of a comprehensive assessment of bone health in the elderly.

In addition, the impact of WWI on bone health has been compared with other obesity indicators, such as BMI and WC [20, 21]. Traditional obesity indices such as BMI and WC often fall short in capturing the nuances of fat distribution and metabolic risk. In contrast, WWI has emerged as a significant anthropometric measure in assessing body composition, particularly in relation to sarcopenia and visceral fat. Several studies have explored the utility of WWI in different populations, highlighting its potential as an integrated index for assessing fat, muscle, and bone health. A study conducted using data from the Korean NHANES demonstrated that higher WWI values are associated with unfavorable body composition outcomes, such as increased fat mass and decreased muscle and bone mass. This study found that WWI was positively correlated with total body fat percentage and inversely correlated with appendicular lean mass and bone mineral density, suggesting that WWI can serve as a comprehensive indicator of body composition [21]. Another study corroborated these findings by showing that WWI positively correlates with total abdominal fat area and visceral fat area, while negatively correlating with appendicular skeletal muscle mass, further supporting its role in reflecting fat and muscle mass in opposite directions in older adults [22].

The menopause-related endocrine shift significantly amplifies the impact of weight and body fat distribution on skeletal health. The withdrawal of estrogen during menopause is well-documented to accelerate bone resorption, which refers to the loss of bone density that occurs due to the increased activity of osteoclasts, the cells responsible for bone breakdown [23, 24]. Concurrently, the hormonal changes associated with menopause lead to fat redistribution toward visceral depots [25, 26]. This phenomenon creates a negative feedback loop: increased visceral adiposity not only exacerbates bone loss by enhancing systemic inflammation and altering hormonal balance but also mitigates the mechanical loading benefits that are usually associated with greater body weight [24, 27]. For instance, the dual pathway hypothesis proposed in some studies suggests that the adipose tissue surrounding the abdomen secretes proinflammatory cytokines and adipokines, which adversely affect bone density [28]. This distinction between visceral and subcutaneous fat is essential when assessing bone health in postmenopausal women, since traditional obesity metrics, such as BMI, often do not account for the specific risks conferred by visceral fat accumulation [29, 30]. Research has shown that visceral fat correlates negatively with BMD, while subcutaneous fat tends to have a neutral or even positive correlation with bone health due to its role as an extraovarian source of estrogen, which can have protective effects against bone resorption [27, 30, 31].

Clinically, our findings suggest that WWI could enhance risk stratification for osteoporosis in postmenopausal women, particularly for normal-weight individuals with central adiposity, who might be overlooked by conventional screening paradigms. Future research should explore whether WWI-guided interventions can mitigate bone loss progression in this vulnerable population. In addition, mechanistic studies investigating WWI's relationship with bone turnover markers and trabecular microarchitecture could further elucidate its pathophysiological role.

This study has several strengths. First, the large, nationally representative NHANES sample enhances generalizability, while rigorous adjustment for confounding variables reduces the likelihood of spurious associations. Second, methodologically, the use of DXA-derived BMD measurements strengthens the clinical relevance of our findings compared to studies relying solely on quantitative ultrasound. In addition, we identified a nonlinear relationship and threshold effect between WWI and BMD using generalized additive models.

However, several limitations warrant consideration. The cross-sectional design precludes causal inference, and residual confounding by unmeasured factors cannot be excluded. Furthermore, WWI's predictive value for fracture outcomes remains to be validated in longitudinal studies. Another limitation of our research is the absence of data for some variables. However, we used contemporary methods to handle the missing data and reduce bias.

## 5. Conclusion

A cross-sectional study of U.S. postmenopausal women revealed a significant inverse correlation between WWI and femoral neck BMD, showing a nonlinear inverted U-shaped relationship with a threshold effect. Further longitudinal studies with larger cohorts are needed to confirm these associations.

## Figures and Tables

**Table 1:** Basic characteristics of participants by weight-adjusted waist index tertile.

Characteristics	Weight-adjusted waist index (cm/√kg)			*p* value
	Low (9.02–11.12)	Middle (11.12–11.78)	High (11.78–14.14)	
	*N* = 1066	*N* = 1066	*N* = 1066	
Age (years)	57.17 ± 10.53	61.49 ± 10.37	65.33 ± 10.31	< 0.001
PIR	3.06 ± 1.66	2.68 ± 1.61	2.32 ± 1.50	< 0.001
Standing height (cm)	162.14 ± 6.47	159.23 ± 6.56	156.63 ± 6.82	< 0.001
BMI (kg/m^2^)	26.41 ± 5.85	29.63 ± 6.51	31.85 ± 6.66	< 0.001
Total femur BMD	0.88 ± 0.14	0.88 ± 0.15	0.86 ± 0.15	0.035
Femoral neck BMD	0.75 ± 0.14	0.73 ± 0.14	0.71 ± 0.14	< 0.001
ALP (IU/L)	70.41 ± 24.51	77.84 ± 25.84	83.53 ± 31.22	< 0.001
BUN (mmol/L)	4.96 ± 1.89	5.26 ± 2.10	5.63 ± 2.31	< 0.001
Globulin (g/L)	29.22 ± 4.60	29.74 ± 4.68	30.45 ± 4.99	< 0.001
Serum glucose (mmol/L)	5.40 ± 1.79	5.93 ± 2.29	6.30 ± 2.68	< 0.001
Triglycerides (mmol/L)	1.30 ± 0.76	1.69 ± 2.29	1.83 ± 1.04	< 0.001
Uric acid	274.07 ± 71.59	301.71 ± 79.78	319.02 ± 80.71	< 0.001
Race (%)				< 0.001
Mexican American	61 (5.72%)	136 (12.76%)	144 (13.51%)	
Other Hispanic	98 (9.19%)	122 (11.44%)	126 (11.82%)	
Non-Hispanic White	451 (42.31%)	408 (38.27%)	473 (44.37%)	
Non-Hispanic Black	270 (25.33%)	246 (23.08%)	183 (17.17%)	
Other race	186 (17.45%)	154 (14.45%)	140 (13.13%)	
Education level (%)				< 0.001
Less than high school	121 (11.35%)	195 (18.31%)	299 (28.08%)	
High school	215 (20.17%)	269 (25.26%)	281 (26.38%)	
More than high school	730 (68.48%)	601 (56.43%)	485 (45.54%)	
Menopause status (%)				< 0.001
No	263 (24.67%)	135 (12.66%)	74 (6.94%)	
Yes	749 (70.26%)	886 (83.11%)	935 (87.71%)	
Other or refused	54 (5.07%)	45 (4.22%)	57 (5.35%)	

*Note:* Mean ± SD for continuous variables: the *p* value was calculated by the weighted linear regression model. Percentage (%) for categorical variables: the *p* value was calculated by the weighted chi-square test. Among the 3198 patients, the amount of missing value for the covariates were 347 (10.9%) for PIR, 3 (0.09%) for standing height, 3 (0.09%) for BMI, 156 (4.9%) for ALP, 156 (4.9%) for BUN, 158 (4.9%) for globulin, 156 (4.9%) for serum glucose, 158 (4.9%) for triglycerides, 157 (4.9%) for uric acid, and 156 (4.9%) for menopause status. ALP, alkaline phosphatase; PIR, income-to-poverty ratio.

Abbreviations: BMD, bone mineral density; BMI, body mass index; BUN, blood urea nitrogen.

**Table 2:** Association between weight-adjusted waist index (cm/√kg) and femoral neck BMD in postmenopausal women.

Exposure	Model 1	Model 2	Model 3
WWI (tertile)	*β* (95% CI) *p* value	*β* (95% CI) *p* value	*β* (95% CI) *p* value
Low (9.02–11.12)	Reference	Reference	Reference
Middle (11.13–11.78)	−0.01 (−0.02, 0.01) 0.4419	−0.01 (−0.02, −0.00) 0.0136	−0.01 (−0.02, −0.00) 0.0029
High (11.79–14.14)	−0.03 (−0.04, −0.01) 0.0001	−0.03 (−0.04, −0.02) < 0.0001	−0.02 (−0.03, −0.01) < 0.0001

*Note:* Model 1: no covariates were adjusted. Model 2: age, race, standing height, and BMI were adjusted. Model 3: age, race, standing height, BMI, total femur BMD, ALP, BUN, globulin, serum glucose, triglycerides, and uric acid were adjusted.

**Table 3:** Threshold effect analysis of WWI (cm/√kg) on femoral neck BMD (g/cm^2^) in postmenopausal women.

WWI (cm/√kg)	Femoral neck BMD
Fitting by the standard linear model	−0.01 (−0.01, −0.01) < 0.0001
Fitting by the two-stage piecewise linear model	
Inflection point	10.32
< 10.32	0.03 (0.00, 0.06) 0.0265
> 10.32	−0.01 (−0.02, −0.01) < 0.0001
Log-likelihood ratio	0.002

*Note:* Age, race, standing height, BMI, total femur BMD, ALP, BUN, globulin, serum glucose, triglycerides, and uric acid were adjusted. The log-likelihood ratio (0.002) indicates significantly improved model fit (*p* < 0.05) when using the two-stage piecewise linear model over the standard linear model, supporting the threshold effect at WWI = 10.32. ALP, alkaline phosphatase.

Abbreviations: BMI, body mass index; BUN, blood urea nitrogen.

**Table 4:** Stratified analyses of the association between weight-adjusted waist index (cm/√kg) and femoral neck BMD.

	*β* (95% CI)	*P* for interaction
Stratified by age		0.4654
40–55	0.01 (−0.00, 0.02) 0.0773	
56–65	−0.00 (−0.01, 0.01) 0.7527	
66–80	−0.01 (−0.02, −0.00) 0.0056	
Stratified by race		0.3257
Mexican American	−0.03 (−0.05, −0.01) 0.0005	
Other Hispanic	−0.03 (−0.05, −0.01) 0.0014	
Non-Hispanic White	−0.02 (−0.03, −0.02) < 0.0001	
Non-Hispanic Black	−0.00 (−0.02, 0.01) 0.8111	
Other race	−0.02 (−0.04, −0.01) 0.0055	
Stratified by standing height (cm)		0.2034
135.3–156.1	−0.01 (−0.02, −0.00) 0.0294	
156.2–162.2	−0.01 (−0.02, 0.00) 0.1349	
162.3–187.8	−0.00 (−0.01, 0.01) 0.5265	
Stratified by BMI (kg/m^2^)		< 0.0001
14.2–25.7	−0.05 (−0.06, −0.04) < 0.0001	
25.8–31.2	−0.05 (−0.06, −0.04) < 0.0001	
31.3–63	−0.06 (−0.07, −0.05) < 0.0001	
Stratified by menopause status		0.0451
No	0.03 (0.01, 0.04) 0.0016	
Yes	−0.01 (−0.02, −0.01) < 0.0001	
Other or refused	−0.06 (−0.08, −0.03) < 0.0001	
Stratified by total femur BMD		0.964
0.362–0.806	−0.02 (−0.02, −0.01) < 0.0001	
0.807–0.93	−0.01 (−0.02, −0.01) < 0.0001	
0.931–1.384	−0.02 (−0.02, −0.01) 0.0003	
Stratified by ALP (IU/L)		0.0613
14–63	−0.03 (−0.04, −0.01) < 0.0001	
64–82	−0.02 (−0.03, −0.01) 0.0002	
83–501	−0.02 (−0.03, −0.01) 0.0027	
Stratified by BUN (mmol/L)		0.2045
0.36–3.93	−0.02 (−0.03, −0.00) 0.0064	
4.28–5.36	−0.01 (−0.02, −0.00) 0.0174	
5.71–22.85	−0.03 (−0.04, −0.02) < 0.0001	
Stratified by globulin (g/L)		0.0849
16–27	−0.03 (−0.04, −0.02) < 0.0001	
28–30	−0.03 (−0.04, −0.02) < 0.0001	
31–65	−0.02 (−0.03, −0.01) < 0.0001	
Stratified by serum glucose (mmol/L)		0.1271
2.61–5	−0.01 (−0.03, −0.00) 0.0185	
5.05–5.55	−0.03 (−0.05, −0.02) < 0.0001	
5.61–32.03	−0.03 (−0.04, −0.02) < 0.0001	
Stratified by triglycerides (mmol/L)		0.0032
0.305–1.061	−0.02 (−0.03, −0.01) 0.0021	
1.073–1.694	−0.03 (−0.04, −0.01) < 0.0001	
1.705–68.384	−0.03 (−0.04, −0.02) < 0.0001	
Stratified by uric acid (μmol/L)		0.0013
41.6–255.8	−0.02 (−0.03, −0.01) < 0.0001	
261.7–315.2	−0.03 (−0.04, −0.02) < 0.0001	
321.2–701.9	−0.03 (−0.05, −0.02) < 0.0001	

*Note:* In subgroup analyses stratified by age, race, standing height, BMI, total femur BMD, ALP, BUN, globulin, serum glucose, triglycerides, and uric acid, the model adjusted for covariates such as age, race, standing height, BMI, total femur BMD, ALP, BUN, globulin, serum glucose, triglycerides and uric acid, but the model did not adjust for the stratification variables themselves. ALP, alkaline phosphatase.

Abbreviations: BMI, body mass index; BUN, blood urea nitrogen.

## Data Availability

Publicly available datasets were used for analysis in this study. The data are located at https://www.cdc.gov/nchs/nhanes/.

## Nomenclature

WWI: Weight-adjusted waist index
BMD: Bone mineral density
PIR: Income-to-poverty ratio
BMI: Body mass index
LDL-C: Low-density lipoprotein cholesterol
HDL-C: Direct high-density lipoprotein cholesterol
AST: Aspartate transaminase
ALT: Alanine transaminase
BUN: Blood urea nitrogen

## Supporting Information

Supporting Table S1. Threshold effect analysis of WWI (cm/√kg) on femoral neck BMD (g/cm^2^) in postmenopausal women.

Supporting Figure S1. Association between WWI (cm/√kg) and femoral neck BMD in postmenopausal women.
