# Supplementary material for: Association Between Weight-Adjusted Waist Index (WWI) and Bone Mineral Density in Postmenopausal Women: A Cross-Sectional Analysis of NHANES Data
Source: Int J Endocrinol. 2025 Sep 27;2025:6618917. doi: 10.1155/ije/6618917 (PMC12496148; doi:10.1155/ije/6618917)
Supplement: Supporting Information — Additional supporting information can be found online in the Supporting Information section. [file 6618917.f1.zip › Supplementary Figure S1.docx]

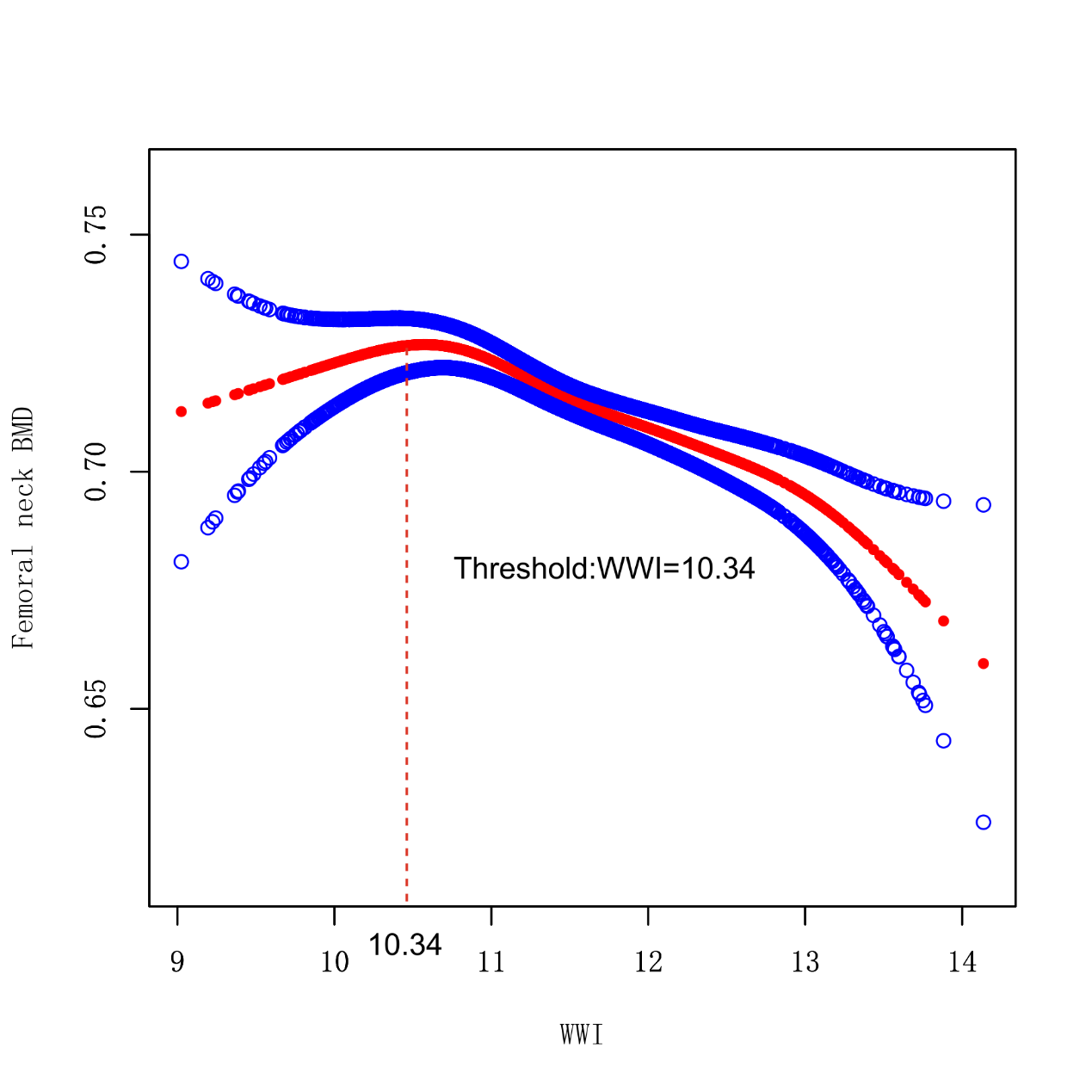


Supplementary Figure S1 Association between weight-adjusted waist index (cm/√kg)

and femoral neck BMD in Postmenopausal Women. The adjustment variable contains

missing data including age, race, standing height, BMI, total femur BMD, ALP, BUN,

globulin, serum glucose, triglycerides, and Uric acid. (The solid red line

indicates the smooth curve fit between variables, and the blue bands denote the 95%

confidence interval from the fit).
