# Supplementary material for: Association Between Weight-Adjusted Waist Index (WWI) and Bone Mineral Density in Postmenopausal Women: A Cross-Sectional Analysis of NHANES Data
Source: Int J Endocrinol. 2025 Sep 27;2025:6618917. doi: 10.1155/ije/6618917 (PMC12496148; doi:10.1155/ije/6618917)
Supplement: Supporting Information — Additional supporting information can be found online in the Supporting Information section. [file 6618917.f1.zip › Supplementary Table S1.docx]

Supplementary Table S1 Threshold effect analysis of WWI (cm/√kg) on femoral neck BMD (g/cm^2^) in postmenopausal women.

| WWI (cm/√kg) | Femoral neck BMD |
| --- | --- |
| Fitting by the standard linear model | -0.01 (-0.01, -0.01) <0.0001 |
| Fitting by the two-piecewise linear model |  |
| Inflection point | 10.34 |
| <10.34 | 0.03 (0.00, 0.05) 0.0221 |
| > 10.34 | -0.01 (-0.02, -0.01) <0.0001 |
| Log likelihood ratio | 0.002 |

Note: The adjustment variable contains missing data including Age, race, standing

height, BMI, Total femur BMD, ALP, BUN, globulin, serum glucose, triglycerides and Uric

acid. BMI, Body Mass Index; ALP, Alkaline Phosphatase; BUN, Blood Urea Nitrogen.

The log-likelihood ratio (0.002) indicates significantly improved model fit (p<0.05) when

using the two-piecewise linear model over the standard linear model, supporting the

threshold effect at WWI=10.34.
