# Supplementary material for: Association Between Weight-Adjusted Waist Index (WWI) and Bone Mineral Density in Postmenopausal Women: A Cross-Sectional Analysis of NHANES Data
Source: Int J Endocrinol. 2025 Sep 27;2025:6618917. doi: 10.1155/ije/6618917 (PMC12496148; doi:10.1155/ije/6618917)
Supplement: Supporting Information — Additional supporting information can be found online in the Supporting Information section. [file 6618917.f1.zip › SupplementaryMaterials.docx]

Supplementary Table S1 Threshold effect analysis of WWI (cm/√kg) on femoral neck BMD (g/cm^2^) in postmenopausal women.

| WWI (cm/√kg) | Femoral neck BMD |
| --- | --- |
| Fitting by the standard linear model | -0.01 (-0.01, -0.01) <0.0001 |
| Fitting by the two-piecewise linear model |  |
| Inflection point | 10.34 |
| <10.34 | 0.03 (0.00, 0.05) 0.0221 |
| > 10.34 | -0.01 (-0.02, -0.01) <0.0001 |
| Log likelihood ratio | 0.002 |

Note: The adjustment variable contains missing data including Age, race, standing

height, BMI, Total femur BMD, ALP, BUN, globulin, serum glucose, triglycerides and Uric

acid. BMI, Body Mass Index; ALP, Alkaline Phosphatase; BUN, Blood Urea Nitrogen.

The log-likelihood ratio (0.002) indicates significantly improved model fit (p<0.05) when

using the two-piecewise linear model over the standard linear model, supporting the

threshold effect at WWI=10.34.


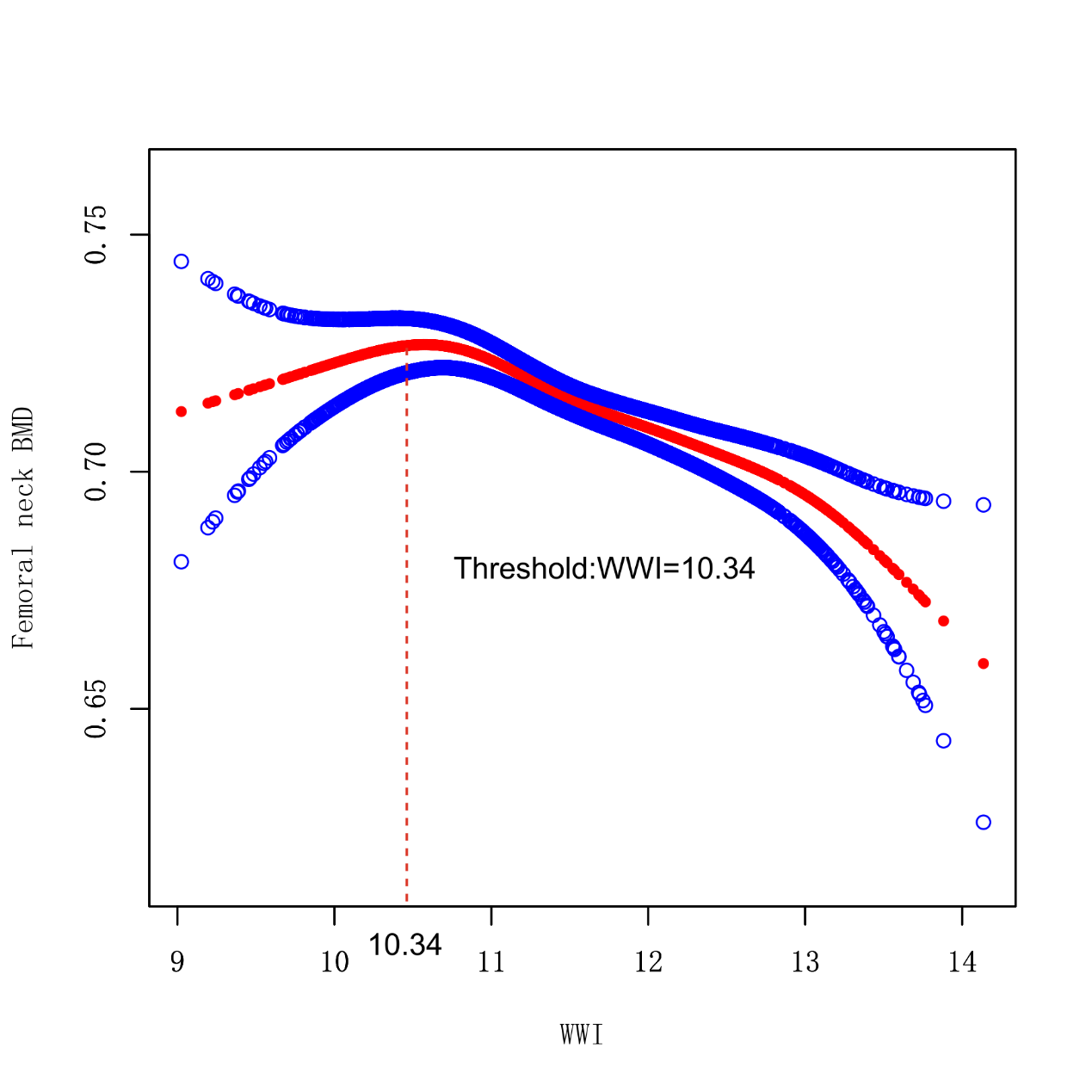


Supplementary Figure S1. Association between weight-adjusted waist index (cm/√kg)

and femoral neck BMD in Postmenopausal Women. The adjustment variable contains

missing data including age, race, standing height, BMI, total femur BMD, ALP, BUN,

globulin, serum glucose, triglycerides, and Uric acid. (The solid red line

indicates the smooth curve fit between variables, and the blue bands denote the 95%

confidence interval from the fit).
